# Supplementary material for: The effect of different irrigation activation techniques on smear layer removal, bioceramic sealer penetration, and interfacial adaptation: SEM and CLSM evaluation
Source: PLoS One. 2025 Oct 9;20(10):e0334063. doi: 10.1371/journal.pone.0334063 (PMC12510540; doi:10.1371/journal.pone.0334063)
Supplement: S1 File — (DOCX) [file pone.0334063.s001.docx]

**Raw data**

**Smear layer**

**G I**

| **Sample** | **Coronal** | **Middle** | **Apical** |
| --- | --- | --- | --- |
| **1** | 2 | 2 | 3 |
| **2** | 1.5 | 4 | 2 |
| **3** | 3 | 2 | 3 |
| **4** | 1 | 2 | 3 |
| **5** | 2.5 | 2.5 | 3 |
| **6** | 2.5 | 2 | 4 |
| **7** | 1.5 | 5 | 5 |
| **8** | 2.5 | 4 | 2 |
| **9** | 1 | 2.5 | 3 |
| **10** | 2 | 2 | 2 |

**G II**

| **Sample** | **Coronal** | **Middle** | **Apical** |
| --- | --- | --- | --- |
| **1** | 2 | 2 | 4 |
| **2** | 1 | 2 | 3 |
| **3** | 2 | 1.5 | 3 |
| **4** | 3 | 2 | 4 |
| **5** | 1 | 2 | 2 |
| **6** | 1 | 2 | 2 |
| **7** | 1 | 3 | 3.5 |
| **8** | 2.5 | 2.5 | 2.5 |
| **9** | 3 | 2 | 2.5 |
| **10** | 2 | 1.5 | 2.5 |

**G III**

| **Sample** | **Coronal** | **Middle** | **Apical** |
| --- | --- | --- | --- |
| **1** | 2.5 | 2.5 | 2 |
| **2** | 2 | 1.5 | 3 |
| **3** | 2 | 3 | 2.5 |
| **4** | 1.5 | 2 | 3 |
| **5** | 1.5 | 1 | 2 |
| **6** | 1 | 3.5 | 2 |
| **7** | 2 | 2.5 | 2 |
| **8** | 3.5 | 2.5 | 2.5 |
| **9** | 2 | 1.5 | 2.5 |
| **10** | 1.5 | 1 | 4.5 |

**G IV**

| **Sample** | **Coronal** | **Middle** | **Apical** |
| --- | --- | --- | --- |
| **1** | 1 | 1 | 2 |
| **2** | 1 | 2 | 3 |
| **3** | 2 | 2 | 2.5 |
| **4** | 1 | 1 | 1 |
| **5** | 2 | 3 | 3 |
| **6** | 1.5 | 2 | 3 |
| **7** | 2 | 2 | 3 |
| **8** | 2 | 2 | 3 |
| **9** | 2.5 | 2.5 | 3 |
| **10** | 2 | 2 | 2 |

**Bioceramic sealer penetration (measurement in µm)**

**G I**

| **Sample** | **Coronal** | **Middle** | **Apical** |
| --- | --- | --- | --- |
| **1** | 88.91 | 269.92 | 115.79 |
| **2** | 154.64 | 192.73 | 79.24 |
| **3** | 111.07 | 227.7 | 98.38 |
| **4** | 171.51 | 149.98 | 101.73 |
| **5** | 61.44 | 81.76 | 111.29 |
| **6** | 92.92 | 137.87 | 114.93 |
| **7** | 327.52 | 202.69 | 108.2 |
| **8** | 101.67 | 71.92 | 87.17 |
| **9** | 212.82 | 171.05 | 92.95 |
| **10** | 152.38 | 135.7 | 95.53 |

**G II**

| **Sample** | **Coronal** | **Middle** | **Apical** |
| --- | --- | --- | --- |
| **1** | 238.41 | 209.76 | 145.23 |
| **2** | 167.2 | 177.38 | 93.88 |
| **3** | 171.32 | 140.98 | 107.52 |
| **4** | 223.34 | 130.5 | 86.51 |
| **5** | 169.41 | 107.13 | 81.44 |
| **6** | 114.87 | 179.55 | 129.68 |
| **7** | 181.5 | 179.1 | 121.15 |
| **8** | 230.3 | 145.84 | 110.65 |
| **9** | 214.05 | 161.69 | 116.52 |
| **10** | 174.56 | 122.93 | 107.79 |

**G III**

| **Sample** | **Coronal** | **Middle** | **Apical** |
| --- | --- | --- | --- |
| **1** | 175.58 | 180.14 | 100.25 |
| **2** | 131.13 | 187.48 | 105.59 |
| **3** | 207.83 | 153.57 | 81.83 |
| **4** | 123.71 | 141.47 | 102.16 |
| **5** | 172.90 | 125.83 | 135.13 |
| **6** | 146.73 | 144.2 | 141.09 |
| **7** | 138.59 | 116.6 | 139.25 |
| **8** | 122.90 | 146.58 | 129.18 |
| **9** | 184.32 | 152.95 | 140.08 |
| **10** | 174.88 | 147.23 | 125.21 |

**G IV**

| **Sample** | **Coronal** | **Middle** | **Apical** |
| --- | --- | --- | --- |
| **1** | 145.18 | 145.22 | 106.38 |
| **2** | 168.52 | 162.45 | 129.56 |
| **3** | 211.64 | 182.07 | 128.62 |
| **4** | 152.72 | 173.63 | 116.98 |
| **5** | 188.86 | 177.62 | 135.83 |
| **6** | 146.01 | 168.93 | 122.67 |
| **7** | 176.18 | 230.96 | 121.61 |
| **8** | 146.78 | 169.38 | 148.37 |
| **9** | 128.09 | 206.11 | 113.98 |
| **10** | 159.18 | 158.93 | 124.66 |

**Interfacial gap (measurement in µm)**

**G I**

| **Sample** | **Coronal** | **Middle** | **Apical** |
| --- | --- | --- | --- |
| **1** | 102.2 | 24.04 | 35.28 |
| **2** | 30.34 | 31.94 | 11.04 |
| **3** | 52.20 | 28.04 | 22.48 |
| **4** | 19.37 | 36.52 | 3.25 |
| **5** | 21.48 | 31.43 | 9.32 |
| **6** | 37.17 | 40.47 | 8.88 |
| **7** | 102.2 | 25.29 | 12.74 |
| **8** | 39.96 | 18.88 | 19.17 |
| **9** | 29.36 | 24.11 | 10.43 |
| **10** | 43.92 | 19.09 | 28.52 |

**G II**

| **Sample** | **Coronal** | **Middle** | **Apical** |
| --- | --- | --- | --- |
| **1** | 40.47 | 41.80 | 54.29 |
| **2** | 78.2 | 64.15 | 26.31 |
| **3** | 60.98 | 46.17 | 42.85 |
| **4** | 21.51 | 16.93 | 24.43 |
| **5** | 43.35 | 28.63 | 31.77 |
| **6** | 15.11 | 10.44 | 50.18 |
| **7** | 50.82 | 39.10 | 20.57 |
| **8** | 19.21 | 28.39 | 35.29 |
| **9** | 24.41 | 10.93 | 25.63 |
| **10** | 20.66 | 31.06 | 30.32 |

**G III**

| **Sample** | **Coronal** | **Middle** | **Apical** |
| --- | --- | --- | --- |
| **1** | 18.89 | 20.95 | 15.94 |
| **2** | 23.28 | 22.13 | 25.23 |
| **3** | 40.62 | 24.84 | 4.97 |
| **4** | 21.87 | 19.91 | 27.88 |
| **5** | 18.39 | 19.09 | 14.63 |
| **6** | 27.1 | 11.38 | 23.59 |
| **7** | 15.76 | 18.95 | 17.98 |
| **8** | 44.00 | 27.75 | 32.45 |
| **9** | 15.3 | 22.53 | 16.56 |
| **10** | 17.85 | 12.38 | 25.45 |

**G IV**

| **Sample** | **Coronal** | **Middle** | **Apical** |
| --- | --- | --- | --- |
| **1** | 9.32 | 18.33 | 26.71 |
| **2** | 39.33 | 15.2 | 15.41 |
| **3** | 31.74 | 25.36 | 44.25 |
| **4** | 23.23 | 21.26 | 17.21 |
| **5** | 25.30 | 32.22 | 30.9 |
| **6** | 28.96 | 9.23 | 42.38 |
| **7** | 15.15 | 28.0 | 29.34 |
| **8** | 28.84 | 8.7 | 47.46 |
| **9** | 26.54 | 16.2 | 24.25 |
| **10** | 17.9 | 20.1 | 29.22 |
