# Supplementary material for: The effect of different irrigation activation techniques on smear layer removal, bioceramic sealer penetration, and interfacial adaptation: SEM and CLSM evaluation
Source: PLoS One. 2025 Oct 9;20(10):e0334063. doi: 10.1371/journal.pone.0334063 (PMC12510540; doi:10.1371/journal.pone.0334063)
Supplement: Attachment — Submitted filename: Response to Reviewers.docx [file pone.0334063.s002.docx]

**Manuscript ID:** PONE-D-25-26888

**Title:** The effect of different irrigation activation techniques on smear layer removal, bioceramic sealer penetration, and interfacial adaptation: SEM and CLSM evaluation

**Authors:** Maryam Saber Mahdi, Ranjdar Mahmood Talabani

**Reviewer #1**

**Comment 1:** Was the samples analysed for curvature and what method was used?

**Response:** Yes, the root canal curvature of all samples was analyzed using the Schneider method, which has now been clearly described in the Materials and Methods section. We have cited Balani et al., 2015 [17] as a reference for this method.

**Comment 2:** Cite recent 2025 article.

**Response:** We have added recent 2025 references to the manuscript and discussion:

• Kilic et al., 2025 [41]

• Sobhy, 2025 [42]

**Comment 3:** Check for grammatical errors.

**Response:** The manuscript has been thoroughly revised for grammar, punctuation, and language clarity to ensure readability and compliance with PLOS ONE standards.

**Comment 4:** Add discussion from 2025 articles.

**Response:** Discussion has been updated to incorporate findings from the 2025 articles [41,42].

**Comment 5:** The study is performed adequately following protocol.

**Response**: We thank the reviewer for the positive evaluation of our methodology.

**Reviewer #2**

**Comment:** In this study, the author(s) compared the effect of different irrigation activation techniques on smear layer removal, bioceramic sealer penetration, and interfacial adaptation using SEM and CLSM evaluation techniques. According to my review, the experimental design and academic writing are very consistent and well-done. It is a very comprehensive article that will contribute to the literature.

**Response:** We thank the reviewer for the positive feedback and acknowledgment of the study’s design, methodology, and contribution to the literature.
